# Prevalence of co-morbid depression in out-patients with type 2 diabetes mellitus in Bangladesh

**DOI:** 10.1186/1471-244X-12-123

**Published:** 2012-08-22

**Authors:** Tapash Roy, Cathy E Lloyd, Masuma Parvin, Khondker Galib B Mohiuddin, Mosiur Rahman

**Affiliations:** 1 BRAC Health Programme, Dhaka, Bangladesh & Division of Research in Medicines and Health, University of Nottingham, Nottingham, UK; 2 Faculty of Health & Social Care, The Open University, Milton Keynes, UK; 3 Medical Education & Research Unit, Ministry of Health, Government of Bangladesh, Dhaka, Bangladesh; 4 School of Business, North South University, Dhaka, Bangladesh; 5 BRAC Health Programme, BRAC Centre (16th Floor), 75 Mohakhali, Dhaka, 1212, Bangladesh

**Keywords:** Diabetes, Depression, Prevalence, Risk factors, Bangladesh

## Abstract

**Background:**

Little is known about the prevalence of depression in people with diabetes in Bangladesh. This study examined the prevalence and factors associated with depression in out-patients with Type 2 diabetes in Bangladesh.

**Methods:**

In this cross-sectional study a random sample of 483 diabetes out-patients from three diabetes clinics in Bangladesh was invited to participate. Of them 417 patients took part. Depressive symptoms were measured using previously developed and culturally standardized Bengali and Sylheti versions of the World HealthOrganization-5 Well Being Index (WHO-5) and the Patient Health Questionairre-9 (PHQ-9) with predefined cut-off scores. Data was collected using two different modes; e.g. standard assisted collection and audio questionnaire methods. Associations between depression and patient characteristics were explored using regression analysis.

**Results:**

The prevalence of depressive symptoms was 34% (PHQ-9 score ≥ 5) and 36% (WHO-5 score < 52) with audio questionnaire delivery method. The prevalence rates were similar regardless of the type (PHQ-9 vs. WHO-5) and language (Sylheti vs. Bengali) of the questionnaires, and methods of delivery (standard assisted vs. audio methods). The significant predictors of depressive symptoms using either the PHQ-9 or WHO-5 questionnaires were; age, income, gender, treatment intensity, and co-morbid cardiovascular disease. Further, depression was strongly associated with poor glycaemic control and number of co-morbid conditions.

**Conclusions:**

This study demonstrated that depression prevalence is common in out-patients with type 2 diabetes in Bangladesh. In a setting where recognition, screening and treatment levels remain low, health care providers need to focus their efforts on diagnosing, referring and effectively treating this important disease in order to improve service delivery.

## Background

Evidence suggests that the prevalance of depression is elevated in those with chronic illnesses such as diabetes [1,2]. Current epidemiological evidence suggests that at least one third of people with diabetes suffer from clinically relevant depressive disorders [3-5]. However, in spite of the huge impact of co-morbid depression and diabetes on the individual and its importance as a public health problem, little is known about the existence of psychological problems in people with diabetes in Bangladesh or the Bangladeshi origin immigrant population in the west [6]; a population group who have a markedly increased risk of developing Type 2 diabetes mellitus (T2DM) [6-8]. Although there is a scant literature describing the prevalence of depression in Bangladesh, there is an assumption that the burden of mental disorders on the Bangladeshi population is high [9]. Limited data from South Asian settings have reported two to five-fold increases in the prevalence rates of depression in people with diabetes compared to people without diabetes [9-12]. One of the major challenges in assessing depression rates in South Asian countries is that no depression screening tools have been culturally standardized for these specific populations. Previous research has demonstrated the potential of a range of different modes of data collection in these ethnic groups (where illiteracy rates are high), including audio versions of questionnaires, as well as assisted completion, depending on the type of questionnaire to be completed [13]. Our recent research in the UK has developed culturally specific methods for administering and collecting reliable data on psychological morbidity in South Asian people with T2DM [14]. The present study set out to screen for the prevalence of and factors associated with the risk of depression in a random sample of out-patients with T2DM living in Bangladesh where literacy problems in data collection also arise, using recently developed and culturally standardized Bengali and Sylheti audio versions of the Patient Health Questionnaire-9 (PHQ-9) and the World Health Organization-5 Well-being Questionnaire (WHO-5).

## Methods

### Study sites

The study was conducted between November 2010 and February 2011. We purposefully selected three sites for data collection, two in urban Dhaka and the other in suburban Sylhet. The three specific sites of data collection were i) the Bangladesh Institute of Research and Rehabilitation in Diabetes, Endocrine and Metabolic Disorders (BIRDEM) Hospital in Dhaka, ii) National Health Care Network facility operated by the Diabetic Association of Bangladesh in Dhaka, and iii) the Diabetic Association of Bangladesh Hospital in Sylhet.

### Subjects

**Figure 1:**
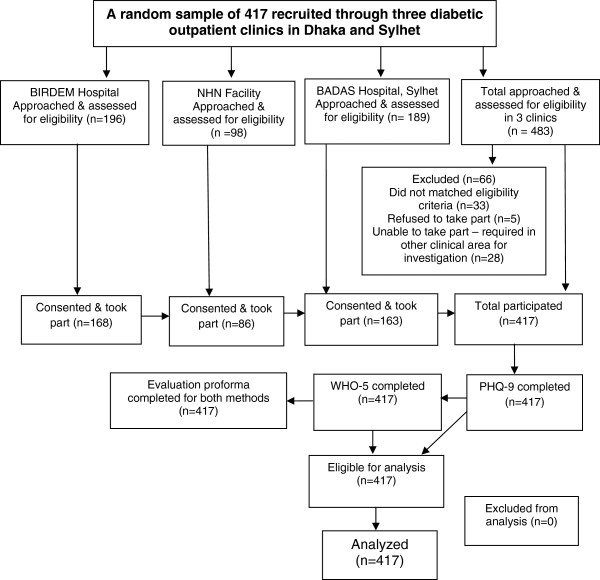
Flow participants through research study.

Individuals who had been diagnosed with T2DM for at least one year, spoke either Bengali or Sylheti and were attending the outpatient department of our selected sites for consultation were approached by the research team and invited to participate in the study. Further inclusion criteria were: (i) age 18–65 years, (ii) capable of independent communication, and (iii) capable of giving informed verbal consent to this study. Individuals who were currently being treated for depression or other psychological problems (e.g. anxiety or personality disorders) as ascertained at recruitment were excluded. In all settings, consecutive attendees in the diabetes outpatient department were randomly approached. Medical officers or resident physicians assisted with recruitment by allowing the researcher to sit in on consultations, during which time the study could be explained, and the patient invited to participate. Figure 1 illustrates the flow of participants through the research study. During this study period, 483 patients attending the three outpatient clinics were approached and invited for initial screening, of them 417 fulfilled the inclusion criteria, provided informed consent and took part in the study.

### Instruments and measurement

Previously developed and evaluated Bengali and Sylheti versions of the PHQ-9 and the WHO-5 were used. Our recent research in the UK has developed culturally specific methods for administering and collecting reliable and valid data on psychological morbidity in South Asian people with T2DM (specifically Mirpuri and Sylheti speakers) and established the cultural equivalency and face validity of two widely used depression screeners [14]. In this latter qualitative study, individuals with T2DM from two minority ethnic populations living in Birmingham, whose main language was only spoken and did not have an agreed written form, participated in a series of focus groups during which both the content and form of delivery of these screening tools designed to measure psychological wellbeing in people with diabetes were evaluated and culturally sensitive written and spoken translations were adopted [14].

The PHQ-9 consists of nine items on a 4-point likert-type scale. It has been shown to have good sensitivity and specificity with regard to identifying cases of depression as well as being sensitive to change over time [15-17]. Standard cut-off scores were used with the PHQ-9 to classify minimal (0–4), mild (5–9), and moderate to severe (≥10) symptoms of depression. The PHQ-9 can be used as a screening tool, with recommended cut-off scores of 10 or greater being found to have 88% sensitivity and 88% specificity for a diagnosis of major depression.

The WHO-5 is a well-validated measure of positive well-being, widely used in a range of settings and has been shown to have good sensitivity to depressive symptoms or depressive affect [18-20]. Unlike most other scales, it is a positive mood scale, measuring the absence rather than the presence of negative mood during the past two weeks. The degree to which these feelings were present in the last 2 weeks is scored on a six-point Likert scale ranging from 0 (not present) to 5 (constantly present). The raw scores are transformed to a score from 0 (worst thinkable well-being) to 100 (best thinkable well-being). A score < 52 suggests poor emotional well-being and is a sign for further testing. A score ≤ 28 is indicative of depression [18,19].

Prior to this study, written and audio versions of both questionnaires were developed in Bengali and Sylheti and evaluated through consultation with a group of Sylheti and Bengali speaking individuals with T2DM attending a diabetes outpatient department in Birmingham, UK [14]. The process of the development of audio version of these questionnaires has been reported elsewhere [14].

### Procedure and evaluation

A research team of 5 members was involved in this study. One of the investigators (TR) and four other trained research assistants (2 male and 2 female; fluent in Bengali/Sylheti) collected the data. A demographic form used in the previous study [14] was modified for use in Bangladesh to assess eligibility. During the selection process in the doctor’s consultation room those who agreed to take part were invited to move to a separate room (education room/counselling room) in the hospital to try out the methods. Female participants were mostly approached and interviewed by female research team members. However, if any male member interviewed them, they were accompanied by either a female member of hospital staff or the person attending the hospital with them. The aims of the study were explained to participants by the researcher and informed consent obtained using pre-validated audio methods [13,14]. Demographic and health related information was collected from each participant. Questions about health related variables included: age of onset & duration of diabetes, body mass index (BMI), fasting blood sugar (FBS), glycosylated haemoglobin (HbA1c) level, medications types, insulin treatment duration, leisure-time physical activity and co-morbidities. The most recent clinical data (e.g. BMI, medication history, FBS, HbA1c level) were collected from the patients’ “personal medical diary” [a handbook with all up to date medical records, which every patient has to carry during follow-up consultation visits] or medical records.

Each session with individual participants consisted of two phases - the first of which was to complete two questionnaires (the PHQ-9 and the WHO-5) using the following modes of data collection:

1. Standard assisted collection (Bengali version), with questions read out by the researcher and answered by the participant with the researcher completing the questionnaire for them.

2. Independent audio collection (either Bengali or Sylheti version), with the participant listening to an audio recording of questions in the appropriate language/dialect, and responding independently using the colour-coded scoring system.

For the second phase of the study and following questionnaire completion, the participants were asked to take part in a brief interview with the researchers in order to discuss their experiences of completing the PHQ-9 and the WHO-5 and to complete an evaluation proforma for each method tested. The evaluation proforma, developed during a previous study [14], was used to assess participants’ opinions on the administration and completion of audio-assisted methods. The detail description about the proforma and evaluation process are published elsewhere before [14].

Ethical approval for the main phase of the study in the UK was obtained via the Birmingham Heartlands Local Research Ethics Committee (now re-configured as part of the Integrated Research Application System [http://www.myresearchproject.org.uk]. The modified protocol for undertaking the study in Bangladesh was reviewed by project team members prior to obtaining ethical approval to carry out the study, from the Bangladesh Medical Research Council (BMRC) and the ethical review committee of BIRDEM, Bangladesh. The investigations were carried out in accordance with the principles of the Declaration of Helsinki as revised in 2000.

### Data analysis

All statistical analyses were conducted using Statistical Package for Social Science version 17 (SPSS Inc., Chicago). Comparisons between groups of subjects were performed using the student’s *t*-test for continuous variables and the Chi- square test for dichotomous variables. The internal consistency of the PHQ-9 and WHO-5 was measured by Cronbach’s α coefficient. Multiple logistic regression was used to assess the adjusted effects of explanatory variables on outcomes. Two separate models were conducted for the PHO-9 and the WHO-5 scores respectively. A forward stepwise method was used to identify the best models with variables to a significance level of 5%. Only statistically significant variables at the univariate level were entered into the models.

## Results

### Demographic and clinical characteristics of the sample

Of the 483 who were invited to participate, 86% (n = 417) completed both questionnaires, of whom 49.4% (n = 206) were female. The mean age of the subjects was 53.2 years. Just under half (45%, n = 189) of the respondents were Sylheti speakers. As shown in Table 1, Sylheti were mostly rural in origin and had lower levels of education compared with Bengali speakers. There was no other significant difference observed between Sylheti and Bengali speakers in terms of demographic, metabolic and clinical characteristics. More than one third of the respondents (35%, n = 145) were on insulin treatment, with 31% (n = 130) reporting high blood pressure and 18% (n = 75) reporting a history of cardiovascular disease. One fifth (20%, n = 83) of study participants reported more than one other co-morbidity.

**Table 1:** Participants according to demographic information and literacy skill

**Category**	**Total Sample**	**Sylheti speakers**	**Bengali speakers**
Total approached for screening (n)	483	220	263
Total recruited and taken part % (n)	86 (417/483)	45 (189/417)	55 (228/417)
Hospital			
BIRDEM, Dhaka % (n)	40.3 (168/417)	21 (35/168)	79 (133/168
NHN, Dhaka % (n)	20.6 (86/417)	14 (12/86)	86 (74/86)
BADAS Hospital Sylhet % (n)	39 (163/417)	87 (142/163)	13 (21/163)
Current Age (Mean ± SD)	53.2 (7.6)	53.9 (8.2)	53 (6.9)
% (n) Female	49.4 (206/417)	47 (89/189)	51 (117/228)
Monthly income in Taka ^a^	9684 ± 6265	9376 ± 6096	9873 ± 6497
% (n) on insulin treatment	35 (145/417)	40 (75/189)	31 (70/228)
Duration of Insulin Treatment (in years) (Mean ± SD)	10.3 (3.9)	10.9 (4.0)	9.8 (3.8)
% (n) Illiterate	42.3 (176/417)	50 (94/189)	36 (82/228)
% (n) rural resident	55.4 (231/417)	79 (149/189)	16 (37/228)
% (n) Sylheti speakers	45.3 (189/417)	NA	NA
Bengali Language Skill			
% (n) Can’t speak, read or write	17 (70/417)	65.4 (67/189)	1 (03/228)
% (n) Can speak only; can’t read or write	17 (72/417)	2 (04/189)	30 (68/228)
% (n) Can read or write only; can’t speak	11 (45/417)	24 (45/189)	-
% (n) Can speak, read and write	55 (233/417)	38.6 (78/189)	70 (157/228)
Treatment intensity			
% (n) Oral medication + diet	65 (272/417)	60 (114/189)	69 (158/228)
% (n) Insulin	19.4 (81/417)	23 (44/189)	16 (37/228)
% (n) Insulin + oral medication	15.3 (64/417)	16 (31/189)	15 (33/228)
% reported leisure-time physical activity	81.3 (339/417	86 (162/189)	78 (177/228)
Type of co-morbidity			
% (n) Cardiovascular Disease ^b^	18 (75/417)	18 (34/189)	18 (41/228)
% (n) Hypertension (SBP >140/DBP > 90 mmHg)	31 (130/417)	37 (70/189)	26 (60/228)
% (n) Others (Kidney/eye problem nephropathy)	4 (18/417)	6.3 (12/189)	3 (06/228)
SBP [mmHg] (Mean *±* SD)	124 (17)	127 (19)	121 (16)
DBP [mmHg] (Mean *±* SD)	78 (11)	79 (13)	76 (10)
BMI (g/cm2) (Mean *±* SD)	24.5 (4.2)	24.7 (4.6)	24.8 (4.5)
% (n) BMI ≤30 g/cm2	68 (283/417)	69.5 (131/189)	71 (161/228)
% (n) BMI > 30 g/cm2	32 (134/417)	30.5 (58/189)	29 (67/228)
FBS level (mg/dl) (Mean *±* SD)	154.7 (57.4)	158.6 (59.3)	152.4 (52.7)
HbA1c level (mmol/mol)(Mean *±* SD)	65 (−8)	67(−7)	65(−9)
Age at Diagnosis (Mean *±* SD)	40 (3.3)	40.3 (3.4)	39.7 (3.1)
Duration of Medication [in years] (Mean ± SD)	13.2 (6.7)	13.6 (7.3)	12.9 (6.3)
Number of medication (Mean *±* SD)	3.8 (1.9)	3.9 (1.9)	3.8 (1.8)
Number of co-morbidity (Mean *±* SD)	1.2 (0.5)	1.3 (0.5)	1.2 (0.5)

^a^One US$ = 84 Bangladeshi Taka.

^b^cardiovascular disease, includes conditions such as coronary heart disease (angina and heart attack) and stroke.

*SD* standard deviation.

*BIRDEM* Bangladesh Institute of Research and Rehabilitation in Diabetes, Endocrine and Metabolic Disorders.

*NHN* National Health Care Network.

*BADAS* Diabetic Association of Bangladesh.

*SBP* systolic blood pressure.

*DBP* diastolic blood pressure.

*BMI* body mass index.

*FBS* fasting blood sugar.

*HbA1c* glycosylated haemoglobin.

### Reliability and item analysis

Cronbach’s α for the PHQ-9 scale was 0.89 and for the WHO-5 it was 0.93. The correlations between nine items of the PHQ-9 and total PHQ-9 scores ranged from 0.71 to 0.83, and all correlations were significant at the 0.01 level. Likewise, the correlations between the five items of the WHO-5 and total WHO-5 scores ranged from 0.73 to 0.89 (with all correlations significant at the 0.01 level) [data not shown in the Table].

### Prevalence of depression

Table 2 shows the mean scores and the proportion of elevated scores on the two measures of depression by method of completion, spoken language and gender of the respondents. The prevalence of depressive symptoms using the PHQ-9 (score ≥5) was 34% (n = 142) when using the audio questionnaire delivery method. When a cut-off value (PHQ-9 ≥10) indicative of moderate to severe depression was used, the prevalence was found to be 16.5% (n = 69). Using the commonly used criteria of the WHO-5 (score < 52, a sign for further testing), 36% of the respondents reported poor well-being when using the audio data collection method. When a lower WHO-5 score (≤ 28) was used, 17.5% of the patients had scores that were suggestive of clinical depression. The prevalence rates of depression were significantly higher in females with T2DM compared with males with T2DM for both screeners (Table 1). The prevalence of depressive symptoms as measured either by the PHQ-9 or the WHO-5 were similar regardless of the language of the questionnaires (Sylheti vs. Bengali) or the method used (the standard assisted vs. independent audio) to complete the questionnaires.

**Table 2:** Prevalence of depression (WHO-5 or PHQ-9) in Sylheti and Bengali speakers with type 2 diabetes mellitus

**Category**	**Audio method**^**b**^**(n = 417)**	**Standard Assisted method**^**c**^**(n = 417)**	**Sylheti speakers (n = 189)**	**Bengali speakers (n = 228)**	**Female (n = 206)**	**Male (n = 211)**
PHQ-9 score (Mean ± SD)	4.1 ± 6.1	4.3 ± 6.2	4.2 ± 6.4	4.0 ± 5.9	5.1 ± 6.3	3.1 ± 5.8
% (n) Depression (sore ≥5-27)	34(142)	33 (137)	36.5 (69)	32 (73)	43 (89) ^a^	25 (53)
% (n) PHQ-9 score 10 or above	16.5 (69)	15.5 (65)	18.5 (35)	14.9 (34)	21.8 (45) ^a^	11.4 (24)
WHO-5 score (Mean ± SD)	54 ± 26	52 ± 27	53 ± 27	56 ± 25	46 ± 23	63 ± 28
% (n) WHO-5 < 52 (poor well-being) ^d^	36 (151)	38 (158)	38.6 (73)	34 (78)	46.6 (96) ^a^	26 (55)
% (n) WHO-5 ≤ 28 (depressive affect)	17.5 (73)	16.8 (70)	19 (36)	16 (37)	22.8 (47) ^a^	12 (26)

^a^p < 0.01comparing female and male with Type 2 diabetes.

^b^Audio method (either Bengali or Sylheti version), with the participant listening to an audio recording of questions in the appropriate language/dialect, and responding independently using the colour-coded scoring system.

^c^Standard assisted method (Bengali version), with questions read out by the researcher and answered by the participant with the researcher completing the questionnaire for them.

^d^using the alternative cut-off value WHO-5 < 50 resulted in the same percentages.

### Factors associated with depression

Table 3 illustrates the results of univariate analysis examining the associations between demographic and clinical factors and depression symptoms (using PHO-9 scores ≥10 and WHO-5 scores ≤ 28). The prevalence of symptoms of depression was more than three times higher in women compared with men for the PHQ-9 (Odds ratio[OR] 3.4; 95% confidence interval [CI] 2.2-5.4) and for the WHO-5 questionnaires; it was 2.7 times higher (OR 2.7; 95% CI 2.0-3.9) in women compared with men. For both the PHQ-9 and the WHO-5, other demographic variables that found statistically significant were low income, older age, lower education level and urban residence. Among the metabolic and clinical variables, patients on insulin or combined insulin and oral treatment, those taking a higher number of medications, those with co-morbid heart disease or a higher number of co-morbidities, and higher BMI, FBS and HbA1c values were all found to be significantly associated with a greater risk for depression, for both questionnaires and both methods of questionnaire completion.

**Table 3:** Univariable regression examining the associations between demographic and clinical factors and depression symptoms (PHO-9 score ≥10 and WHO-5 score ≤ 28)

**Variables**	**PHQ-9**	**WHO-5**
	**OR (95% CI)**	**OR (95% CI)**
Current Age (r = lowest value, 41 years)	1.8 (1.3-2.2) ^b^	1.7 (1.0-2.5) ^b^
Female (r = male)	3.4 (2.2-5.4) ^b^	2.7 (2.0-3.9) ^b^
Schooling in year (r = 0)	0.7 (0.5-1.6) ^c^	0.6 (0.4-1.5) ^c^
Monthly Income > 6000 Taka (r = ≤ 6000 Taka) ^a^	0.4 (0.3-0.9)^b^	0.5 (0.3-1.1) ^b^
Urban resident (r = rural)	1.6 (1.0-2.8) ^c^	1.5 (0.9-4.2) ^c^
Treatment Centre (r = BADAS Hospital Sylhet)	1.3 (0.7-3.8)	1.2 (0.6-3.5)
Sylheti Speaker (r = Bengali speaker)	1.1 (0.6-2.4)	1.0 (0.5-3.1)
Method of data collection (r = audio collection method)	1.0 (0.5-2.3)	1.1 (0.6-3.3)
Treatment intensity		
Insulin (r = oral medication + diet)	1.4 (1.0-2.8) ^c^	1.5 (1.1-3.2) ^c^
Insulin + oral medication (r = oral medication + diet)	1.7 (1.0-2.9) ^c^	1.8 (1.0-3.4) ^c^
Duration of Medication (r = lowest value, 2 years)	1.1 (0.7-3.3)	1.2 (0.6-3.8)
Number of medication (r = lowest number, 3 medicines)	1.5 (0.8-2.2) ^c^	1.4 (0.8-2.4) ^c^
Co-morbidity		
Cardiovascular Disease (r = no co-morbidity)	1.8 (1.2-2.5) ^b^	2.1 (1.5-3.2) ^b^
Hypertension (r = no co-morbidity)	1.2 (0.5-3.9)	1.1 (0.5-3.7)
Kidney/eye problem/nephropathy (r = no co-morbidity)	1.0 (0.5-3.4)	1.0 (0.6-3.5)
Number of co-morbidity (r = 0)	2.2 (1.4-3.5) ^b^	2.4 (1.5-3.4) ^b^
BMI (r = lowest value, 21.3 g/cm2)	0.7 (0.4-1.1) ^c^	0.6 (0.3-1.2) ^c^
FBS level (r = lowest value, 97.3 mg/dl)	1.8 (1.1-2.5) ^b^	1.6 (1.1-3.1) ^b^
HbA1c level (r = lowest value, 40 mmol/mol)	2.1 (1.7-2.8) ^b^	2.4 (1.5-3.1) ^b^

^a^One US$ = 84 Bangladeshi Taka.

^b^P value <0.01.

^c^P value <0.05; r = reference category.

*WHO-5* World Health Organization-5 Well Being Index.

*PHQ-9* Patient Health Questionairre-9.

*OR* odds ratio.

*CI* confidence interval.

Table 4 presents the results of the multivariate logistic regression, identifying the significant independent predictors of depression symptoms (using PHO-9 score ≥10 and WHO-5 score ≤ 28). For both models (PHQ-9 and WHO-5); female sex, lower income, older age, patients with combined insulin and oral therapy, co-morbid heart disease and a higher number of co-morbidities were all found to be independent predictors for depression. Poor glycaemic control (as indicated by high FBS and HbA1c values), was also revealed as a strong predictor for depression in both models (Table 4). In both multivariate models, number of medications and obesity (indicated by BMI > 30.0 kg/m2) were not associated with depressive symptoms after controlling for other factors.

**Table 4:** Multivariate logistic regression predicting depression symptoms (PHO-9 score ≥ 10 and WHO-5 score ≤ 28) by demographic characteristics, metabolic risk factors and diabetes complications

**Variables**	**PHQ-9**	**WHO-5**
	**OR (95% CI)**	**OR (95% CI)**
Current Age (r = lowest value, 41 years)	1.5 (1.0-1.8) ^c^	1.6 (1.1-2.0) ^c^
Female (r = male)	2.8 (2.0-4.8) ^b^	2.3 (1.8-3.7) ^b^
Schooling in year (r = 0)	0.9 (0.6-2.8)	0.8 (0.4-2.5)
Monthly income > 6000 Taka (r = ≤ 6000 Taka) ^a^	0.5 (0.3-1.0) ^b^	0.6 (0.4-1.2) ^b^
Urban resident (r = rural)	1.3 (0.7-3.8)	1.2 (0.6-3.5)
Treatment intensity		
Insulin (r = oral medication + diet)	1.2 (0.8-2.6)	1.2 (0.7-3.4)
Insulin + oral medication (r = oral medication + diet)	1.6 (1.0-2.8) ^c^	1.5 (1.0-3.1) ^c^
Number of medication (r = lowest number, 3 medicines)	1.1 (0.8-3.2)	1.2 (0.8-3.8)
Co-morbidity		
Cardiovascular Disease (r = no co-morbidity)	1.6 (1.1-2.3) ^b^	1.8 (1.3-3.3) ^b^
Hypertension (r = no co-morbidity)	1.1 (0.5-3.8)	1.1 (0.4-3.5)
Kidney/eye problem/nephropathy (r = no co-morbidity)	1.0 (0.5-3.4)	1.0 (0.6-3.5)
Number of co-morbidity (r = 0)	1.8 (1.2-3.3) ^b^	2.1 (1.3-3.7) ^b^
BMI (r = lowest value, 21.3 g/cm2)	0.9 (0.3-1.9)	0.8 (0.2-2.1)
FBS level (r = lowest value, 97.3 mg/dl)	1.6 (1.0-2.8) ^c^	1.5 (1.0-2.9) ^c^
HbA1c level (r = lowest value, 40 mmol/mol)	2.0 (1.4-3.3) ^b^	2.1 (1.3-3.7) ^b^

^a^One US$ = 84 Bangladeshi Taka.

^b^P value <0.01.

^c^P value <0.05.

The prevalence of depression used as dependent variable in the logistic regression analysis was derived from the standard assisted data collection or the audio data collection.

## Discussion

This study examined the prevalence of and factors associated with depression in a random sample of out-patients with T2DM in Bangladesh. To our knowledge, this is the first study that has investigated the prevalence of depression using data collected through two different modes; i.e. standard assisted collection and audio questionnaire methods. We found similar prevalence rates of depressive symptoms regardless of which screening tool was used (PHQ-9 vs. WHO-5), and regardless of the language of the questionnaires (Sylheti vs. Bengali) or the method used (the standard assisted vs. independent audio method).

Our study provides evidence that depression is common in T2DM in Bangladeshi settings. More than one-third of individuals reported poor emotional well-being using the WHO-5 questionnaire and similar prevalence rates of depressive symptoms in patients with T2DM when using the PHQ-9. When using a lower cut-off value for the WHO-5 (score ≤ 28) or a cut-off value for the PHQ-9 (score ≥10) indicative of moderate to severe depression, the prevalence rates of depression were very similar for both questionnaires. These prevalence rates are in the line with recent studies that have used PHQ-9 in the primary care settings [21,22]. This prevalence of poor wellbeing and depressive affect is also comparable to the results of one recent study in diabetic out-patients that reported poor wellbeing in 35–38% and depressive affect in 18–25% of patients with T2DM [19]. A handful of studies have reported a higher prevalence of depression in people with T2DM compared with those without diabetes or in the general population [23-25]. In a systematic review of cross-sectional prevalence data, Ali et al. [2] also reported significantly higher rates of depression in those with T2DM compared to adults without.

An earlier population-based study in Bangladesh has reported almost similar rates of depressive symptoms (29.7%) in a rural population with diabetes using the Montgomery-Asberg Depression Rating Scale [9]. In that earlier study higher socio-economic status and a high BMI were found to be protective factors against depression. We also observed these links in our current study population in Bangladesh, however this association was no longer significant at the multivariate level when controlling for other demographic and clinical variables.

The earlier study further reported that depression was associated with poverty and the authors assumed that as poverty is more prevalent in rural areas, the prevalence of depression may also be higher [9]. Our sample comprised a mix of urban and rural populations and although we found a similar link between poverty and depression, in fact the opposite association was observed in terms of participants’ area of residence. At the univariate level urban residents with T2DM were nearly twice as likely to be depressed as rural residents when measured using either the PHQ-9 or WHO-5 questionnaires. However, this association no longer persisted after controlling for other confounding factors.

Consistent with the results of other published studies in Bangladesh and elsewhere [2,9,23], our results demonstrated a significantly higher prevalence of depression in women with T2DM compared with men with T2DM. After controlling for potential confounding factors, gender remained as the strongest risk factor for depressive symptoms, with nearly a threefold increase risk in females compared with males.

Poor glycaemic control was also a strong predictor of depression in our sample for both multivariate models. This finding is in the line with previous studies [23]. It is known that depression has a negative impact on quality of life and that depression worsens glycaemic control [5,26]. Numerous studies, overwhelmingly cross-sectional, support our findings and suggest that depression is associated with suboptimal glycaemic control, although in a systematic review the effect size was mild [5]. A recent prospective study demonstrated a clear prospective association between depression at baseline and persistently higher HbA1c levels over a 4 year period [27].

Our findings also suggest that the presence of one or more complications, in particular cardiovascular disease, was significantly associated with depression in patients with T2DM. This finding is in the line with recent studies that show that the risk of depression is significantly associated with the number of diabetes-related complications [21,28]. Having multiple chronic diseases in addition to diabetes has a high impact on well-being, quality of life and functioning and thus may contribute to further development of depression [29]. It is suggested that patients with severe diabetes-related complications, especially late micro- and macro-vascular complications such as retinopathy, nephropathy, neuropathy or cardiovascular disease, are more likely to be referred to specialized clinics. If these patients are adequately treated, they are more satisfied with their care and overall functioning [28]. Our findings are relevant for clinicians and nurses who work in diabetes outpatient clinics in order to take timely decisions for appropriate referral.

Evidence also suggest that diabetes complications and depression often coexist and the prevalence of depression is particularly increased in those with longer lasting T2DM, but not in undiagnosed T2DM or those with impaired glucose metabolism [30]. A number of studies highlighted that the incidence of depression is increased in T2DM [31,32] and that depression is a risk factor for T2DM [33]. However, It is important to increase our understanding of the temporal relationship between the development of secondary complications and the onset or recurrence of depression. Future studies should aim to address these issues.

One of the major challenges in measuring depression in Bangladesh is that no depression screening tools have previously been culturally standardized for the population in Bangladesh. Our previous research in the UK has developed culturally specific methods for administering and collecting reliable and valid data on psychological morbidity in South Asian people with T2DM (including Bengali and Sylheti speakers) and has established the face validity and cultural equivalency of two widely used depression screeners (the PHQ-9 and WHO-5) [14]. This research used those culturally standardized tools and demonstrated their utility as potential depression screeners in wider sample like current study population.

A number of studies documented that depression symptomatology is influenced by social and cultural factors [14]. In contrast, this study gives us the impression that the prevalence rates and the risk factors for depression in Bangladesh are very similar to European countries and the US [19,22,23,26]. Thus, it provides a room for argument that even if the meaning of depression varies cross-culturally, its crude prevalence or association to risk factors may not be culture specific.

The findings of this study have major implications for clinical practice in Bangladesh, where physicians’ recognition of mental disorder rates is low and improving recognition rates is a challenge because of the high patient loads and poor undergraduate training in these skills. Providing the patients with the results of blood sugar, cholesterol, blood pressure and medications plan through outpatient service is not enough itself to improve service delivery and bring about change [34]. We need to overcome therapeutic inertia and low diabetes health literacy [35]. There is increasing recognition that patients with diabetes and depression require adequate mental healthcare, however, evidence in favour of routine screening and monitoring is not conducive yet. A few studies have tested whether screening for depression or monitoring of psychological well-being has beneficial effects, but results of these studies are conflicting to come to any conclusion [36-39].

In the developed world (for example in the North America and UK), self management is available for all new cases of diabetes. Undoubtedly, the patients with co-morbid diabetes and depression in Bangladesh would benefit from this approach. In the Bangladeshi settings, the clinician who sees the patients with diabetes could take on the role of initial assessment for depression and coordinate referral to mental health clinic for therapy and onward referral and follow-up with the patients [40].

### Strengths and limitations of the study

The strengths of our study include a high response rate and the inclusive nature of our research as individuals could participate regardless of literacy level. Including patients from two different ethnic backgrounds in Bangladesh was a further strength. Rather than having to rely on self-report, we were able to use information from patients’ medical diaries to gather information about diabetes, glycaemic control and the presence of diabetes complications. Also, a reasonable sample size and ascertaining depression with culturally standardized questionnaires are strengths of this study.

However, an important limitation of our study was that we did not use a psychiatric diagnostic interview such as the Composite International Diagnostic Interview (CIDI), which is considered as the gold standard for the diagnosis of depression. Although it is suggested that the PHQ-9 can be used as a diagnostic assessment in primary care settings, however, the gold standard is still a diagnostic interview and a PHQ-9 diagnosis is regarded as inferior to the diagnostic interview.

In addition, no information was available on the sample’s use of antidepressants, pain scores or daily living activities. This could bias the results, as patients who take antidepressants may have a low PHQ-9 or WHO-5 scores.

## Conclusion

In conclusion, our study demonstrated that depression is a common co-morbid health problem in T2DM out-patients in Bangladesh, with more than one-third of patients reporting elevated depression scores regardless of depression screeners and data collection methods used. Within this sample of out-patients with diabetes, we found that female gender, older age, low income, treatment with combine insulin and oral medications, poorly controlled T2DM, and those with coexisting complications of diabetes were independent risk factors for depression symptoms.

This study provides rich data on the prevalence and determinants of depression in T2DM outpatients in Bangladesh. In a setting where recognition, screening and treatment levels remain low, health care providers need to focus their efforts on diagnosing, referring and effectively treating this important disease in order to deliver rights-based and client-centred services for people in real needs.

## Competing interests

The authors declare that they have no competing interests.

## Authors’ contributions

CEL conceptualized and designed the study. TR collected data, conducted statistical analyses and prepared the manuscript. All authors made significant contributions to the conception and design of the analyses, interpretation of the data, and drafting of the manuscript, and all authors approved the final manuscript.

## Pre-publication history

The pre-publication history for this paper can be accessed here:

http://www.biomedcentral.com/1471-244X/12/123/prepub
